# Supplementary material for: Genetic Screening of a Hungarian Cohort with Focal Dystonia Identified Several Novel Putative Pathogenic Gene Variants
Source: Int J Mol Sci. 2023 Jun 28;24(13):10745. doi: 10.3390/ijms241310745 (PMC10341391; doi:10.3390/ijms241310745)
Supplement: Supplementary file 1 [file ijms-24-10745-s001.zip › ijms-2457225-supplementary.pdf]

Supplementary file – Table S1: List of variants of uncertain significance (VUS) in 30 genes examined in our focal dystonia population

Gene	Transcript number	cDNA position	Protein position	Zygosity	MAF in the gnomAD database among non-Finnish Europeans	ACMG classification	phyloP100way score	Focal dystonia form
ANO3	NM_031418.4	c.2276-6T>C	-	het	0.0062%	VUS	-0.004	CD
ATM	NM_000051.3	c.497-9_497-4delTTTTTT	-	het	0	VUS	0.813	CD
ATM	NM_000051.3	c.7475T>G	p.Leu2492Arg	het	0.024%	VUS	7.549	BSP
ATP7B	NM_000053.3	c.3892G>A	p.Val1298Ile	het	0.0044%	VUS	7.564	CD
ATP7B	NM_000053.4	c.1759A>C	p.Thr587Pro	het	0	VUS	2.425	CD
ATP7B	NM_000053.3	c.2491G>A	p.Val831Ile	het	0.0016%	VUS	2.568	CD
ATP7B	NM_000053.3	c.677G>A	p.Arg226Gln	het	0.0018%	VUS	0.608	BSP
CACNA1A	NM_001127222.1	c.266A>G	p.Lys89Arg	het	0	VUS	7.632	CD
CACNA1A	NM_001127222.1	c.1213G>A	p.Ala405Thr	het	0	VUS	7.026	CD
CACNA1A	NM_001127222.2	c.4150A>G	p.Met1384Val	het	0	VUS	4.063	CD
CACNA1A	NM_001127222.2	c.5668G>A	p.Val1890Ile	het	0	VUS	7.707	CD
CACNA1A	NM_001127222.2	c.6775C>T	p.Arg2259Trp	het	0.0029%	VUS	0.607	CD
CACNA1B	NM_000718.4	c.3287-3T>G	-	het	0	VUS	-1.002	CD
CACNA1B	NM_000718.4	c.265A>G	p.Lys89Glu	het	0	VUS	7.914	CD, CD, BSP, CD, BSP

CACNA1B	NM_000718.4	c.2204T>C	p.Leu735Pro	het	0	VUS	6.107	BSP
CACNA1B	NM_000718.4	c.2848G>A	p.Ala950Thr	het	0	VUS	0.443	CD
CACNA1B	NM_000718.4	c.6950C>T	p.Thr2317Ile	het	0.0125%	VUS	2.169	BSP
CACNA1B	NM_000718.4	c.836G>A	p.Gly279Asp	het	0	VUS	2.384	CD
CACNA1B	NM_000718.4	c.3196C>T	p.Arg1066Cys	het	0.0009%	VUS	4.472	CD
CACNA1B	NM_000718.4	c.5945G>A	p.Arg1982Gln	het	0.0074%	VUS	0.085	CD
CACNA1B	NM_000718.4	c.1079C>G	p.Ala360Gly	het	0	VUS	7.784	CD
CIZ1	NM_001131016.2	c.1820A>G	p.Glu607Gly	het	0	VUS	4.452	CD
COL6A3	NM_004369.3	c.5387C>T	p.Ala1796Val	het	0.0023%	VUS	4.302	CD
COL6A3	NM_004369.3	c.3852C>G	p.Phe1284Leu	het	0.045%	VUS	8.882	CD, CD
COL6A3	NM_004369.3	c.8627C>T	p.Pro2876Leu	het	0.0029%	VUS	2.136	CD
COL6A3	NM_004369.3	c.6902T>C	p.Val2301Ala	het	0.0101%	VUS	2.886	CD, CD
COL6A3	NM_004369.3	c.9508G>C	p.Gly3170Arg	het	0.0045%	VUS	1.163	BSP
COL6A3	NM_004369.3	c.254G>A	p.Gly85Glu	het	0.0054%	VUS	1.912	BSP
COL6A3	NM_004369.3	c.3040A>G	p.Lys1014Glu	het	0.0434%	VUS	2.799	CD
COL6A3	NM_004369.3	c.3817G>A	p.Val666Ile	het	0.0109%	VUS	2.594	BSP, CD
COL6A3	NM_004369.3	c.4510C>T	p.Arg1504Trp	het	0.0712%	VUS	3.274	CD
COL6A3	NM_004369.3	c.6224C>T	p.Pro2075Leu	het	0.0114%	VUS	6.019	BSP
DRD5	NM_000798.4	c.101C>A	p.Pro34Gln	het	0.0025%	VUS	0.082	BSP
KMT2B	NM_014727.2	c.3136C>T	p.Arg1046Cys	het	0	VUS	3.599	CD

KMT2B	NM_014727.2	c.6784C>G	p.Leu2262Val	het	0	VUS	0.191	CD
KMT2B	NM_014727.2	c.127G>T	p.Val43Leu	het	0	VUS	2.511	BSP
KMT2B	NM_014727.2	c.676C>T	p.Arg226Trp	het	0	VUS	0.129	BSP
LRRK2	NM_198578.4	c.6867G>C	p.Lys2289Asn	het	0.0009%	VUS	5.804	BSP
LRRK2	NM_198578.4	c.3451G>A	p.Ala1151Thr	het	0.0093%	VUS	2.372	BSP
REEP4	NM_025232.4	c.538C>T	p.Arg180Trp	het	0.0176%	VUS	0.465	CD
REEP4	NM_025232.4	c.734G>A	p.Arg245Gln	het	0.0156%	VUS	1.895	BSP
SPR	NM_003124.5	c.193C>T	p.Arg65Trp	het	0.0065%	VUS	-0.002	BSP
SYNE1	NM_182961.4	c.11594C>T	p.Thr3865Met	het	0.0093%	VUS	0.959	BSP
SYNE1	NM_182961.4	c.17848G>T	p.Val5950Leu	het	0.0088%	VUS	7.487	BSP
SYNE1	NM_182961.4	c.25051C>G	p.Gln8351Glu	het	0	VUS	9.051	CD
SYNE1	NM_182961.4	c.17650C>T	p.Pro5884Ser	het	0.0155%	VUS	7.703	CD
SYNE1	NM_182961.4	c.12056C>A	p.Ala4019Glu	het	0.0053%	VUS	9.54	CD
TH	NM_000360.3	c.599G>A	p.Arg200His	het	0.0127%	VUS	3.717	BSP
TH	NM_000360.3	c.67G>A	p.Ala23Thr	het	0.0274%	VUS	0.813	CD
VPS16	NM_022575.4	c.1370T>C	p.Leu457Pro	het	0	VUS	7.103	CD
VPS16	NM_022575.4	c.2501C>T	p.Ala834Val	het	0	VUS	3.124	CD
VPS16	NM_022575.4	c.1136C>G	p.Ala379Gly	het	0.0071%	VUS	7.317	CD
VPS41	NM_014396.4	c.1391T>C	p.Ile464Thr	het	0.1148%	VUS	7.517	CD, CD

Abbreviations: ACMG - American College of Medical Genetics and Genomics; *ANO3* – anoctamin 3; *ATM* – ataxia-telangiectasia mutated; *ATP7B* – ATPase copper transporting beta; BSP - benign essential blepharospasm; *CACNA1A* – calcium channel, voltage-dependent, P/Q type, alpha-1A subunit; *CACNA1B* - calcium channel, voltage-dependent, N type, alpha-1B subunit; CD – cervical dystonia; *CIZ1* – CIP1-interacting zinc finger protein; *COL6A3* – collagen, type VI, alpha-3; *DRD5* – dopamine receptor D5; het – heterozygous; *KMT2B* – lysine-specific methyltransferase 2B; *LRRK2* – leucine-rich repeat kinase 2; MAF – minor allele frequency; *REEP4* – receptor expression-enhancing protein 4; *SPR* – sepiapterin

reductase; *SYNE1* – spectrin repeat-containing nuclear envelope protein 1; *TH* – tyrosine hydroxylase; *VPS16* – VPS16 core subunit of corvet and HOPS complexes; *VPS41* – VPS41 subunit of HOPS complex
